# Association between Nutritional Status, Lifestyle Habits, and Disease Activity in Dalmatian Patients with Rheumatoid Arthritis

**DOI:** 10.3390/nu15071738

**Published:** 2023-04-01

**Authors:** Mislav Radić, Ivan Vlak, Marijana Vučković, Josipa Radić, Erim Bešić, Tonko Vlak

**Affiliations:** 1 Internal Medicine Department, Rheumatology, Allergology, and Clinical Immunology Division, Center of Excellence for Systemic Sclerosis in Croatia, University Hospital of Split, 21000 Split, Croatia; 2 Department of Internal Medicine, School of Medicine, University of Split, 21000 Split, Croatia; 3 Institute of Physical Medicine and Rehabilitation with Rheumatology, University Hospital Split, Šoltanska 1, 21000 Split, Croatia; 4 Internal Medicine Department, Nephrology and Haemodialysis Division, University Hospital of Split, 21000 Split, Croatia; 5 Faculty of Pharmacy and Biochemistry, Department of Biophysics, University of Zagreb, 10000 Zagreb, Croatia; 6 Department of Physical and Rehabilitation Medicine, University of Split, School of Medicine, 21000 Split, Croatia

**Keywords:** rheumatoid arthritis, nutritional risk, muscle mass, muscle strength, disease activity

## Abstract

The aim of this study was to evaluate body composition, handgrip strength, quality of life, disease duration and activity and lifestyle habits in patients with rheumatoid arthritis (RA) and to evaluate possible associations between all of the abovementioned factors. Seventy-five stable RA patients were included. Data on sociodemographic data, disease activity, quality of life, nutritional risk, body mass composition, anthropometric parameters, and clinical and laboratory parameters were collected for each study participant. The results showed that the mean score of the disease activity score (DAS28) was 5.4, the mean score of the health assessment questionnaire-disability index (HAQ-DI) was 1.19, and the mean disease duration in our population was 13.9 years. Our studied population had a long disease duration and high disease activity. Positive predictors of muscle mass in RA patients were daily caloric intake, fat-free mass, bone mass, basal metabolic rate, total body water, weight, body mass index (BMI), height, and muscle strength. There were no significant negative predictors. Positive predictors of muscle strength in RA patients were daily caloric intake, basal metabolic rate, predicted muscle mass, fat-free mass, bone mass, weight, total body water, metabolic age, hemoglobin, BMI, and number of exercises per week. In contrast, negative predictors of muscle strength were number of comorbidities, number of swollen joints, DAS, number of tender joints, erythrocyte sedimentation rate (ESR), and duration of RA. An association was also found between bone mineral density and both muscle mass and muscle strength. A structured nutritional approach in terms of multidisciplinary collaboration between rheumatologist, dietitian and physical medicine specialist is needed in the Dalmatian RA population.

## 1. Introduction

Rheumatoid arthritis (RA) is a chronic, systemic, progressive, autoimmune disease of unknown etiology and female predominance with an impact on various aspects of the lives of affected patients, from psychosocial, physical, systemic, and in general, quality of life as a multidimensional concept. Chronic inflammation, joint affection with potential development for irreversible changes over time, and reduced muscle strength affects mostly the physical part of patients, with extraarticular manifestations and a systemic preference for many organic systems. In comparison to general population depression, RA patients are doubly more prone to cognitive impairment and anxiety. With a higher disease activity, there is a higher degree of anxiety and depression in RA population connecting physical and psychological components. The studies also showed lower a socio-economic status in this group of patients with all the mentioned components forming an unbreakable connection and vicious circle of rheumatoid arthritis [1,2,3,4]. Its prevalence rate in European adults ranges from 3.1 to 8.5 per 10,000 cases, which makes it the most prevalent rheumatic inflammatory disease [5]. Increased overall cardiovascular risk (including myocardial infarction, cerebrovascular accidents, and congestive heart failure) in comparison to the general population is 48% [6], and the risk of infections is higher due to disease pathophysiology and immunomodulatory therapy and has the potential for being even higher depending on chronic comorbid conditions of the patients or their lifestyle factors [7]. This makes these patients a particularly vulnerable population. The studies have shown that glucocorticoid therapy is a major risk factor among immunosuppressive drugs [7]. Another increased risk in RA patients is that osteoporosis is more significant in patients who are older, physically more disabled, have a higher disease activity, and with longer disease duration, as well being seropositive RA patients [8]. There is also a higher risk for overfat, obesity, and sarcopenia which are observed even at the early stage of disease. Female patients have a higher prevalence of sarcopenia and sarcopenic obesity, with them also being the group with a lower percentage of lean mass, making them more vulnerable compared to male patients [9,10,11]. Distribution and proportions of lean mass and fat mass have important health implications, not only in the group of RA patients, but also in the general population. Lower lean mass can lead to cachexia, resulting in greater weakness, higher disability status, and higher mortality and development of other metabolic abnormalities. Higher percentages of fat mass may predispose to arterial hypertension, diabetes, and a higher risk of developing cardiovascular diseases [9]. There is also a connection between higher disease activity (joint deformity, CRP levels, rheumatoid factor seropositivity, lack of treatment) and abnormal body composition [11].

As with many other diseases, risk factors for RA can be divided into nonmodifiable and modifiable. Common modifiable risk factors for the development and worsening of RA include smoking, alcohol and coffee consumption, obesity, and educational level. Smoking has shown to be the most consistent risk factor for RA. The risk is dose related and stronger in males and for anti-citrullinated peptide antibody-positive RA patients. Obesity in seronegative RA patients and high coffee consumption in general may increase RA risk [12]. It has been found that adherence to certain dietary habits can lead to better outcomes, but further studies are necessary for a better understanding and the future implementation of these facts [13,14,15]. Only regular physical activity is part of the international guidelines for the treatment of RA [16]. Physical activity training has shown many benefits, including reversing rheumatoid cachexia, reducing cardiovascular risk, reducing the risk of colon and breast cancer, reducing risk of falls, and improving overall function and strength of patients. Exercise in general improves overall function in RA patients without any proven detrimental effects to disease activity. Most of RA patients have a low physical activity, but it is a reversible characteristic with good therapeutic results for all stages of the disease, making it a necessary area of focus for the future research of training through inflammatory flares. Unfortunately, studies have also shown that most of the beneficial muscle adaptations are lost after cessation of the exercise training [17]. Data on the nutritional status of RA patients are inadequate in some aspects.

People with RA are primarily discouraged from vigorous physical activity due to joint inflammation, swelling, and limited mobility. The inevitable corticosteroid therapy (CS), with its known side effects of infection risk, muscle wasting, increase in adipose tissue, increased appetite, and risk of osteoporosis [18] places this group of patients in a particularly vulnerable position in terms of the risk of developing sarcopenia, with a prevalence of 31% [19]. Many studies have shown that older age (>60 years), presence of a disability, longer disease duration, and malnutrition in patients are positively associated with sarcopenia [20,21], while the use of bDMARDs in some studies is being negatively associated or described without association with these symptoms [20,22]. The use of CSDMARD was negatively associated with sarcopenia in RA patients [22].

Long-term chronic use of nonsteroidal anti-inflammatory drugs (NSAIDs) and CS carries the risk of gastric ulceration and gastrointestinal (GI) bleeding. The use of NSAIDs and CS is associated with a four-time increased risk of a GI adverse effect compared with the use of either drug alone [18]. It can lead to the worsening of the anemia, which is known as one of the comorbidities of chronic RA disease. The results of some studies suggest severe disease joint activity in RA patients with anemia and also a good therapeutic response of the joint disease if the anemia is successfully treated [23]. Nausea, vomiting, and constipation are known side effects of opioid analgesics commonly prescribed for RA patients [24], with constipation being the most common, which in turn can lead to a loss of appetite, decrease in food intake, and consequently, an imbalance of macro- and micronutrients. Some studies demonstrate that use of TNF inhibitors might be associated with a gain in fat mass, while the use of tocilizumab might be associated with a gain in lean mass [9].

Another important factor contributing to the burden of RA are increased levels of oxidative stress and systemic inflammation, which in addition to the classic effects, also affect the balance between muscle recovery and muscle wasting. Inflammatory cytokines are responsible for muscle wasting and stimulating protein catabolism, but the association between sarcopenia and inflammatory parameters is still poorly understood. Sarcopenic patients had significantly higher CRP levels, while IL6 and TNF-α values were not higher in comparison with the control group. Despite the current literature, there is still a need for future studies [25].

Lower muscle mass in the general population is associated with poorer quality of life [26], higher cardiovascular risk [27], dementia, and depression [28]. These two studies included older people (65 years or older) with the diagnosis of sarcopenia and confirmed the connection between depression and cognitive impairment among healthy older men with sarcopenia, and also a lower health-related quality of life for people with a moderate or severe risk of nutritional deficiency [26,28]. More then 50% of rural elderly people in the study were malnourished [28]. Some studies in patients suffering from RA suggest that sarcopenia is related to worse cardiometabolic risk, disease activity [29], disease duration, and age [19].

Since the diagnostic and treatment options are evolving with time, there are better diagnostic approaches, greater knowledge, and more efficient therapeutic options, which lead to the gradual increased survival rate of RA patients, a better control of disease activity, more preventable long-term known musculoskeletal defects, and systemic manifestations, all of which leading to a greater importance of understanding the risk factors, comorbidities, lifestyle habits, and nutritional status of patients for an even better future of treatment and results [30,31].

The aim of this study was to evaluate body composition, handgrip strength, quality of life, disease duration, and activity and lifestyle habits in Dalmatian patients with RA and to evaluate their possible correlations.

## 2. Materials and Methods

This cross-sectional study included 75 stable RA patients and was conducted at the Department for Rheumatology, Clinical University of Split in 2017.

The study was approved by the Ethics Committee of Clinical University Hospital Split. Informed consent was obtained from each study participant.

We excluded patients who had limb amputation, pacemaker, active infection, had serious cognitive impairment, or were unwilling to participate.

### 2.1. Clinical and Laboratory Parameters

By a thorough examination of patients’ medical history data regarding the duration of RA illness, the presence of arterial hypertension, diabetes mellitus, chronic kidney disease, hyperlipidemia, and gastric ulcer were collected for each participant. Data regarding medication usage were also obtained and medications were divided into the following groups: NSAIDs, conventional synthetic disease-modifying anti-rheumatic drugs (CSDMARD), biological disease-modifying anti-rheumatic drugs (BDMARD), paracetamol/tramadol, CS, and usage of 3 or more medications.

Regarding laboratory parameters, data with regard to erythrocyte sedimentation rate (ESR), C-reactive protein, and hemoglobin were collected.

### 2.2. Sociodemographic Data

Using a sociodemographic questionnaire, data regarding education level, marital status, and employment were collected. Data regarding type, duration, and frequency of exercising were also collected for each study participant.

### 2.3. Body Composition and Anthropometric Parameters

Body composition was assessed for every participant using the device (body composition analyzer Tanita BC-1000), which uses bioimpedance analysis (BIA) to determine resistance to different tissues. Data about fat percentage, fat mass (kg), visceral fat level, metabolic age, fat-free mass, total body water, predicted muscle mass, bone mass (kg), basal metabolic rate, and daily calorie intake were obtained. Height was measured using a stadiometer. Body mass index (BMI) was calculated. Handgrip strength was assessed using a hand-held dynamometer (Saehan, [32]).

### 2.4. Disease Activity and Quality of Life Assessment

Disease activity score-(DAS28), which uses the number of tender joints, number of swollen joints, ESR, and a general health assessment (VAS), was used to calculate a quantifiable disease activity index presented as a total score [21,33]. The health assessment questionnaire disability index (HAQ-DI) questionnaire consisting 20 questions in eight categories of functioning—dressing, rising, eating, walking, hygiene, reach, grip, and usual activities—were used to measure health-related quality of life (HrQoL) information for each participant. Scores of 0 to 1 are generally considered to represent mild to moderate difficulty, 1 to 2, moderate to severe disability, and 2 to 3, severe to very severe disability [22,34].

### 2.5. Nutritional Risk Assessment

For a nutritional risk assessment, short-tool nutrition risk screening 2002 (NRS-2002) was used. NRS score ranges from 0 to 7 and NRS score ≥ 3 indicates nutritional risk. This screening tool takes age, BMI, food intake, weight loss and illness severity into consideration [23,35].

### 2.6. Bone Mineral Density (BMD) Assessment

Bone density scanning dual-energy X-ray absorptiometry (DXA scan) was performed for every participant at the Department of Endocrinology, University Hospital Center Split. BMD was measured using Hologic QDR 4500 C (S/N 48034; Bedford, MA 01730, USA) Bone Densitometer, and results were expressed as BMD (g/cm^2^).

### 2.7. Statistical Analysis

Data were analyzed using Minitab 17 statistical software. Comparisons of muscle strength and muscle mass between patients who exercised regularly and patients who did not exercise at all were assessed using a t-test for two independent means assuming equal variances. Analyses of variance (ANOVA) and post hoc Tukey test were used to compare differences in muscle strength and muscle mass between the three groups of patients (osteoporosis, osteopenia, and normal BMD). A multiple linear regression analysis was used to explore the independent correlates of muscle strength/muscle mass total scores (the dependent variable) adjusted for age and sex. Significance level was set at 0.05 (two-tailed tests).

## 3. Results

This cross-sectional study included 75 RA patients, of whom 8.3% were men, and the mean age was 65.75 years. The mean duration of RA disease was 13.9 years. Descriptive statistics of the measured variables are shown in Table 1.

**Figure 1:**
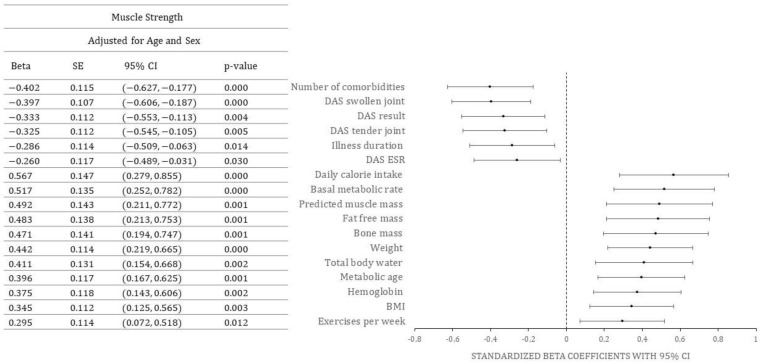
Predictors of muscle strength. Abbreviations: SE-standard error, CI-confidence interval, DAS-disease activity score, DAS ESR-disease activity score erythrocyte sedimentation rate, BMI-body mass index (kg/m^2^).

Standardized beta coefficients for the effect of various predictors on muscle strength, adjusted for age and sex, are shown in Figure 1 (only statistically significant values are shown).

Positive predictors of muscle strength in RA patients were daily caloric intake, basal metabolic rate, predicted muscle mass, fat free mass, bone mass, weight, total body water, metabolic age, hemoglobin, BMI, and number of exercises per week. In contrast, negative predictors of muscle strength were number of comorbidities, number of swollen joints, DAS, number of tender joints, ESR, and duration of RA.

**Figure 2:**
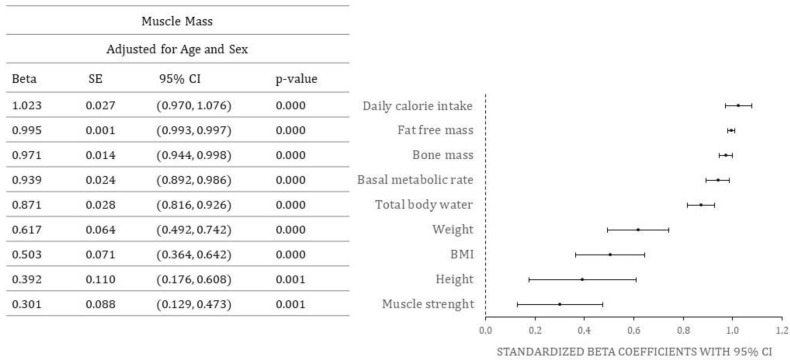
Predictors of muscle mass. Abbreviations: SE-standard error, CI-confidence interval, body mass index (kg/m^2^), CI-confidence interval.

Standardized beta coefficients for the effect of various predictors on muscle mass, adjusted for age and sex, are shown in Figure 2 (only statistically significant values are shown).

Positive predictors of muscle mass in RA patients were daily caloric intake, fat free mass, bone mass, basal metabolic rate, total body water, weight, BMI, height, and muscle strength. There were no significant negative predictors.

**Figure 3:**
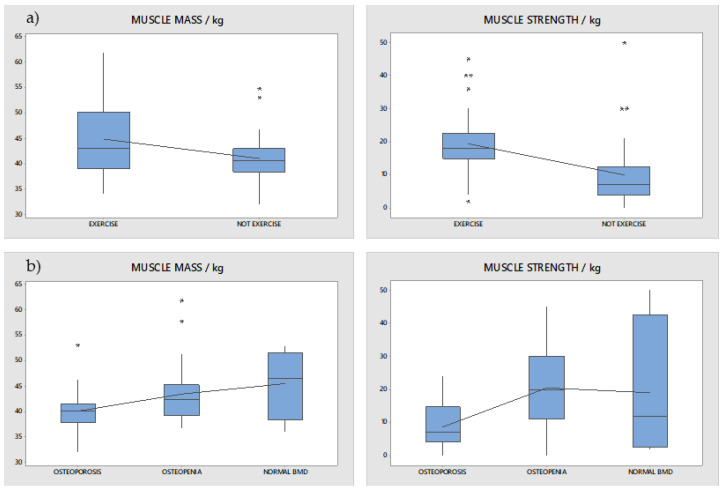
Differences in muscle mass and muscle strength: (**a**) between patients that performed exercises and patients that did not perform exercises (*p* = 0.010); (**b**) between patients with osteoporosis, osteopenia, and normal BMD (*p* = 0.000). Asterisks represent mild outliers (data points that are more extreme than Q1 − 1.5 IQR or Q3 + 1.5 IQR).

Differences in muscle strength and muscle mass between patients who exercised regularly and patients who did not exercise at all were examined using a t-test for two independent means assuming equal variances. It was shown that patients who exercised regularly have a statistically significant higher muscle mass and muscle strength than patients who did not exercise at all (*p* = 0.010, Figure 3a). Additionally, differences in muscle strength and muscle mass between the three groups of patients (osteoporosis, osteopenia, and normal BMD) were compared with ANOVA and post hoc Tukey test. It was shown that patients with osteoporosis have a statistically lower muscle mass and muscle strength compared to patients with osteopenia and normal BMD (*p* = 0.000, Figure 3b).

## 4. Discussion

To our knowledge, this is the first study in our region to investigate the relationship between nutritional parameters, muscle strength, disease activity, body composition, and lifestyle in patients with RA.

Regarding general parameters, it is important to mention that the average value of DAS28 in our studied population is 5.4, and as DAS28 being greater than 5.1 implies active disease, the average value of HAQ-DI was 1.19, which is considered to represent moderate to severe disability (HAQ-DI score 1 to 2). In addition to that, average duration of disease in our population was 13.9 years, which is rather long. Our sample thus represents a group with long disease duration and a highly active RA, which should be taken into consideration when discussing our results.

Regarding nutritional parameters, our results suggest positive associations between daily caloric intake, basal metabolic rate, muscle mass, fat free mass, weight, and muscle strength in RA patients with highly active and long duration disease, which is actually expected and self-explanatory.

Our results suggest that BMI is a positive predictor of both muscle strength and muscle mass in RA patients, which is consistent with the findings of Hye-Won et al., who found that BMI is associated with low muscle mass in RA patients, according to the EWGS definition [24,36]. It is known from the literature that obese individuals are prone to have a constant low-grade inflammation [37], and as our study population is mainly obese, this could be a confounding factor. Additionally, due to our study design, it is not possible to conclude whether nutritional status had an impact on disease severity or vice versa.

Regarding disease activity, DAS proved to be a negative predictor of muscle strength in our study. In a study of 335 RA patients, DAS28 score was positively associated with body fat mass and DAS28-P score was positively associated with body fat skeletal muscle ratio in female RA patients [25,38]. In contrast, our results showed that DAS had no predictive power for muscle mass in a population of RA patients with highly active and long duration disease. The possible explanation for these results could be difference in measurement of muscle mass since it is not evident from the mentioned research which method was used. An additional difference in longer disease duration and a higher DAS28 score in our study when compared to the aforementioned study could imply a greater impact of disease activity on muscle strength in highly active RA, rather than impact on body fat content. Furthermore, a systematic review and meta-regression analysis by Li et al. found both HAQ-DI and DAS score to be predictive of sarcopenia in RA population [19,26]. Our results regarding the negative association between DAS score and muscle strength point to the same direction. With regard to HAQ-DI, we found no significant associations with muscle mass nor muscle strength in our study. Only one study from the mentioned review by Li et al. had DAS28 and HAQ scores as high as the ones from our study [27,39].

Moreover, the number of swollen and tender joints proved to be a negative predictor of muscle strength in our study in a population of RA patients with highly active and long duration disease. This could be explained by the fact that swelling or tenderness in the joint area may limit the mobility and physical activity of RA patients, leading to a vicious cycle that is reflected in lower muscle strength.

One of the negative predictors of muscle strength found in our study was the duration of RA. This result is similar to the finding of a previous study by Torii et al., who found that a longer duration of RA was positively associated with sarcopenia [20,28]. On the other hand, a study by Barone et al. found no association between sarcopenia risk and disease duration in patients with RA, psoriatic arthritis, and ankylosing spondylitis [21,29].

As for comorbid diseases, we found a higher number of comorbidities to be predictive of lower muscle strength in RA, which is consistent with the findings of Gong et al., who found an independent association between comorbidity and skeletal muscle mass/physical performance in hospitalized elderly people [30,40].

Regarding laboratory parameters, our results suggest that Hb level is a positive predictor of muscle strength, and ESR level is a negative predictor of muscle strength in RA patients.

Data on the relationship between Hb level and muscle strength in RA patients are inconclusive [23,31], but in a study of patients with hematologic malignancies, the low-hemoglobin group of participants had a significantly lower muscle strength than the high-hemoglobin group [32,41].

In another study of 730 participants from the I-Lan longitudinal aging study, hemoglobin levels were found to be significantly associated with faster gait speed and stronger handgrip strength. Anemia was also found to be significantly associated with sarcopenia, weakness, and slowness. Stronger correlations between anemia and sarcopenia were found in men and in individuals with severe disease burden [42].

Further prospective studies are needed to better understand this problem in RA patients, but the results of our study may provide an indication of the importance of the management of anemia and its associations with muscle strength and sarcopenia. A study by Barone et al. in patients with RA, psoriatic arthritis, and ankylosing spondylitis found no association between ESR and an increased risk of sarcopenia [21,29], which is in contrast to our findings. The difference of populations in regard to mean disease duration—which is higher in our study than in a subset of RA patients in a study by Barone (13.9 years vs. 10.8 years), and a high proportion of RA patients in remission regarding DAS28 in a study by Barone (47.3%), compared to a high disease activity reported in our study—could be the reason for different findings.

Our results also suggest that a higher number of exercises per week predicts higher muscle strength in RA patients. It is well known from the literature that physical activity has specific health benefits for RA patients, such as improved cardiovascular health, increased muscle mass and muscle strength, reduced adiposity, and improved physical performance [17,33].

A study from Sul et al. found beneficial effects from 12 weeks of upper- and lower-limb strengthening exercise on lower-limb strength and mental health in RA patients [43].

Association between bone mineral density and both muscle mass and strength was found in our study, implying that long duration and highly active RA patients comorbid with osteoporosis or osteopenia had lower levels of both muscle mass and muscle strength. This could be due to the better flexibility which could lead to a lower osteoporosis rate. Another explanation could be that greater disease activity requires higher CS doses, which in turn have an impact on bone mineral density and muscle mass. Unfortunately, due to the design of our study, we are unable to determine causal relations, so more prospective studies are needed to address this question more thoroughly.

Interestingly, no medication use was found to be a statistically significant predictor of muscle mass or muscle strength in our study, although a review by Dao et al. showed that use of CS was positively associated with sarcopenia, and use of CSDMARD was negatively associated with sarcopenia in RA patients [22,34]. It is important to notice that in the mentioned review article from Dao et al., mean DAS28 varied from 2.7 (±1.1) (Remission-low disease activity) to 4.9 (±1.3) (moderate-high disease activity) which is rather lower than DAS28 in our study (5.4). Another explanation for these differences might be the relatively small number of participants and the fact that we did not consider the dosage of certain drugs when conducting this study. Limitations of our study relate primarily to the cross-sectional design, which prevented us from establishing causal relationships among the parameters studied. Another limitation stems from the fact that we did not consider dietary intake when conducting this study.

In conclusion, the specificity of the RA population, characterized by long disease duration and high disease activity, particularly enriches the scope of our study.

## 5. Conclusions

Long standing disease duration and a higher disease activity negatively affect muscle strength in rheumatoid arthritis (RA) patients. There is a positive association between daily caloric intake, basal metabolic rate, muscle mass, fat free mass, weight, and muscle strength in RA patients. Higher number of exercise sessions per week predicts higher muscle strength based on the study findings. The results of the study suggest that body mass index (BMI) is a positive predictor of muscle strength in RA patients, whereas the number of comorbidities is a negative predictor. RA patients with osteoporosis and osteopenia have lower levels of muscle mass and strength; however, due to the study design, causal relationships could not be determined.

## Figures and Tables

**Table 1:** Descriptive statistics of measured variables.

Variable	N	Mean	SD
Age (years)	72	65.75	1.10
BMI (kg/m^2^)	72	26.19	0.54
Illness duration (years)	72	13.90	1.09
Body composition and muscle strength			
Fat percentage (%)	72	36.50	1.03
Fat mass (kg)	72	27.23	1.24
Visceral fat level	72	9.68	0.33
Metabolic age (years)	72	64.68	1.48
Fat free mass (kg)	72	45.16	0.79
Total body water (%)	72	31.47	0.64
Muscle mass (kg)	72	42.87	0.75
Bone mass (kg)	72	2.30	0.04
Basal metabolic rate (cal/day)	72	5705.6	97.5
Daily calorie intake (kJ)	72	9420	176
Muscle strength—men (kg)	6	19.17	1.22
Muscle strength—women (kg)	66	13.95	1.44
Disease specific parameters			
DAS tender joint	72	11.44	0.75
DAS swollen joint	72	7.86	0.49
DAS ESR (mm/hr)	72	28.60	2.53
DAS VAS	72	50.35	2.01
DAS result	72	5.40	0.14
HAQ-DI	71	1.19	0.52
NRS	71	0.33	0.10
Medication			
NSAID	46		
CSDMARD	62		
BDMARD	6		
Paracetamol	4		
Paracetamol/Tramadol	22		
Corticosteroids	55		
Number of medications			
≤3	20		
>3	50		
Comorbidities			
Arterial hypertension	46		
Diabetes mellitus	8		
Chronic kidney disease	1		
Hyperlipidemia	11		
Coronary artery disease	11		
Gastric ulcer	23		
Thyroid disease	15		
Mineral bone density			
Osteopenia	23		
Osteoporosis	36		
Lifestyle			
Education level			
Elementary school	32		
High school	36		
Bachelor’s/Master’s	4		
Martial status			
Married/Widowed	38		
Unmarried	34		
Exercise	34		
N. of weekly exercises			
2	7		
3	9		
4	5		
5	6		
6	4		
7	3		

Abbreviations: N—number, SD—standard deviation, BMI—body mass index (kg/m^2^), DAS—disease activity score, DAS ESR—disease activity score erythrocyte sedimentation rate, DAS VAS—disease activity score visually analogue scale, NRS—nutrition risk screening score, HAQ-DI—health assessment questionnaire-disability index, NSAID—non-steroid anti-inflammatory drug, CSDMARD—conventional synthetic disease-modifying antirheumatic drugs, BDMARD—biologic disease-modifying antirheumatic drugs.

## Data Availability

Data are available by the corresponding author upon e-mail request.
